# Using Recycled Construction Waste Amended with Pine Bark as a Substrate for Urban Plantings

**DOI:** 10.3390/plants15030403

**Published:** 2026-01-28

**Authors:** Claire Kenefick, Stephen J. Livesley, John P. Rayner, Claire Farrell

**Affiliations:** School of Agriculture, Food and Ecosystem Sciences, Burnley Campus, The University of Melbourne, 500 Yarra Boulevard, Richmond, VIC 3121, Australia; sjlive@unimelb.edu.au (S.J.L.); jrayner@unimelb.edu.au (J.P.R.); c.farrell@unimelb.edu.au (C.F.)

**Keywords:** alkalinity, crushed concrete, crushed rock, growing media, recycled substrate, sand, scoria, substrate properties, urban greening, woody plants

## Abstract

In urban plantings, mined sand and scoria are commonly used as low-nutrient substrates to improve plant establishment and growth. However, reliance on mined materials conflicts with sustainability policies promoting resource circularity and waste reuse. Construction wastes are readily available, and while their high alkalinity and density may limit plant growth, incorporating organic matter, like pine bark, can ameliorate these issues. We investigated whether construction waste amended with pine bark can support plant growth. We evaluated physical and chemical properties of 12 substrates combining four mineral components—scoria (mined), sand (recycled), crushed concrete (recycled), and crushed rock (recycled)—with pine bark (10%, 20%, and 50% *v*/*v*). We then tested eight of these substrates in a container experiment, evaluating the growth of two woody shrubs: *Alyogyne huegelii* and *Goodenia ovata*. All mineral components were alkaline (pH 9.2–12.3), with crushed concrete remaining hyper-alkaline despite pine bark addition. Greater pine bark volumes improved air-filled porosity but reduced water retention. Substrates with 50% *v*/*v* pine bark had lower plant biomass compared to those with 10% *v*/*v*. However, plant biomass was similar across all mineral components. This demonstrates that construction waste–pine bark substrates can support plant growth in urban plantings, supporting broader sustainability goals in cities.

## 1. Introduction

Naturalistic plantings can be a low-maintenance approach to increase the amenity and biodiversity of informal green spaces, such as streetscapes and railway easements [1]. They are designed to mimic the structure of natural vegetation communities, layering diverse understorey plant species to create a complex structure with abundant and prolonged flowering [2]. A key design feature of naturalistic plantings is including species that can establish and persist with low inputs [3,4]. However, establishing naturalistic plantings on urban soils can be challenging, as they are often degraded and compacted [5,6].

Urban soils can be highly fertile due to excessive or long-term fertiliser application and runoff [7], which can lead to high weed competition [8]. Urban soils can be improved via (i.) amendments (e.g., gypsum, lime, compost) to improve chemical and physical structure [9,10], (ii.) mechanical ripping or tillage to reduce dominant weed cover, reduce compaction and improve physical structure [6,11,12], or (iii.) a combination of tillage and deep soil amendments [12,13,14]. However, these treatments can be impractical at larger scales and may not address the issues of persistent weed seed banks [6]. In such instances, the options are to remove upper soil layers or to add new upper soil layers [4].

Topsoil removal (or “scalping”) removes the upper 10–50 cm of soil layers containing excess nutrients and weed propagules [15] and has been used for grassland restoration in Australia [16,17], Europe [18,19,20], and North America [21]. However, in urban landscapes, scalping can be limited by the high cost of topsoil removal and disposal [22] or by contaminants that prohibit removal or disturbance of soil [23]. In these cases, new soil layers can be added on top of existing site soil as a “capping substrate”, providing an uncompacted, low-nutrient, weed-free growing medium, and acting as a mulch to suppress weeds [9,24].

Capping substrates are commonly used in naturalistic plantings to mimic free-draining, low-nutrient natural soils, favouring the establishment of target species over nutrient-demanding weeds [8]. To support plant establishment and growth, capping substrates must provide adequate air, water, and structure [25,26,27]. There are currently no guidelines for capping substrates for naturalistic plantings, but acceptable ranges for substrate physical and chemical properties (Table 1) can be drawn from guidelines for horticultural container media [28,29,30,31], green roof substrates [32,33,34,35], and landscape soils [9,36,37].

Capping substrates are typically composed of mined or quarried materials such as sand, gravel, or scoria, due to their low-nutrient content, minimal weed seed load, and well-characterised physical properties [8,40]. However, with a growing desire to reduce the environmental impact of cities, more sustainable materials are required—particularly those that reuse waste generated in cities [41].

Mineral materials recycled from construction and demolition waste could be used as capping substrates for naturalistic plantings. Construction wastes include materials such as crushed concrete, crushed rock, recycled sand, crushed brick, crushed roof tile, and subsoil [39]. They can have lower environmental and economic cost compared to mined materials, so they could be sustainable replacements for existing mineral substrates [42,43]. Previous studies have evaluated construction wastes as the inorganic substrate component for green roofs [44,45,46,47] and in technosols for tree or garden plantings, e.g., [48,49,50,51,52]. However, their use as capping substrates for naturalistic plantings is limited [2,8,22,24]. Construction wastes are generally low in nutrients and may contain fewer weed seeds [22], which would make them ideal capping substrates. However, they can have high bulk density (e.g., recycled sands or subsoils; [39,53]), poor water-holding capacity (e.g., crushed rock; [22]), and extreme alkalinity (e.g., crushed concrete; [47,54]).

Adding organic matter to construction wastes could ameliorate unfavourable physical and chemical properties [55], potentially bringing them into the acceptable ranges for plant growth. Organic matter can increase air and water capacity, decrease density, improve the structure [46,56], and reduce the pH of alkaline construction wastes [54]. There are many types of organic matter, and their origin and pre-treatment (e.g., ageing, composting) can determine the maturity, cellulose-to-lignin ratio, nutrient content, and stability (i.e., rate of decomposition and longevity; [57,58]). For capping substrates, organic matter should be stable with low nutrients and weed seed load. Aged pine bark is a stable organic matter commonly used in soil-less container media and as landscape mulch, due to its favourable physical and chemical properties, low weed seed content, and slow decomposition [25,57,59]. Pine bark is a softwood material produced as a waste by-product from the timber industry and is carbon-rich with low nitrogen availability [57]. Pine bark has been shown to increase air-filled porosity (AFP), water-holding capacity (WHC), and plant available water (PAW), as well as reduce bulk density (BD) in sand container media [60]; it is also acidic [61], which could help ameliorate high alkalinity in construction waste substrates. Pine bark can ameliorate highly alkaline substrates by replacing alkaline mineral particles on a volume-to-volume basis (i.e., physical dilution) as well as through chemical reactions.

We aimed to evaluate the use of construction and demolition mineral wastes with and without pine bark addition as capping substrates for naturalistic plantings. We evaluated the physical, hydrological, and chemical properties of four mineral components—scoria, recycled sand, crushed concrete, and crushed rock—pine bark, and 12 substrate mixes made of the four mineral components with three rates of pine bark addition (10, 20, or 50% *v*/*v*). Based on their properties, we then evaluated plant growth in eight substrates with 10% or 50% pine bark in a nursery-based container experiment. To compare plant response across a range of substrate alkalinity, we selected two drought-tolerant species from low-nutrient Australian shrublands that have demonstrated vigorous growth in woody naturalistic plantings with scoria capping substrates [62] and have contrasting alkalinity tolerance. *Alyogyne huegelii* is found naturally across a range of soil types from acidic to alkaline [63], whereas *Goodenia ovata* is found naturally on neutral to acidic soils [64]. We hypothesised that

1. Greater rates of pine bark addition to mineral substrates will improve physical and hydrological properties and result in greater plant growth as compared to mixes with lower rates of pine bark addition.
2. Greater rates of pine bark addition to alkaline substrates will improve chemical properties and result in greater growth of alkaline-sensitive plant species (*G. ovata*) than alkaline-tolerant species (*A. huegelii*).

## 2. Results

### 2.1. Effect of Pine Bark Addition on Substrate Properties

**Figure 1:**
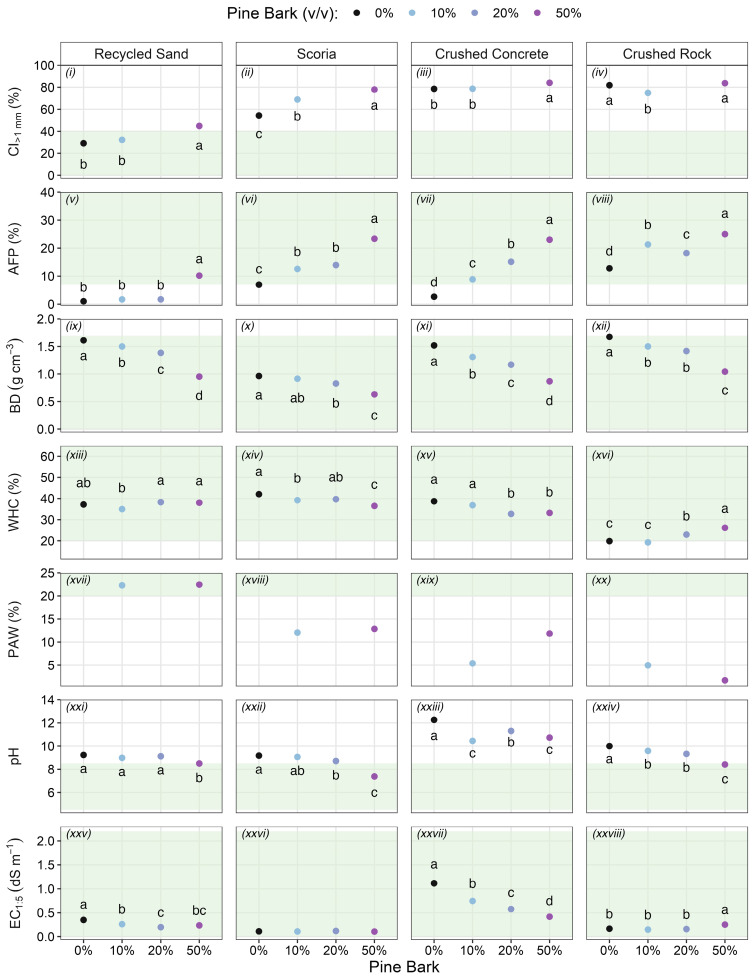
Effects of pine bark addition on physical properties: (**i**–**iv**) coarseness index, (CI_>1 mm_), (**v**–**viii**) air-filled porosity (AFP), (**ix**–**xii**) dry bulk density (BD); hydrological properties: (**xiii**–**xvi**) water-holding capacity (WHC), and (**xvii**–**xx**) plant available water (PAW); and chemical properties: (**xxi**–**xxiv**) pH, and (**xxv**–**xxviii**) electrical conductivity (EC_1:5_). Columns represent the four mineral components: recycled sand, scoria, crushed concrete, and crushed rock. The X-axis and colours represent pine bark rate *v*/*v* (0% = black, 10% = light blue, 20% = grey-blue, and 50% = purple). Values represent means (*n* = 3) for AFP, BD, WHC, pH, and EC; (*n* = 2) for CI; and (*n* = 1) for PAW. Green shading indicates the acceptable range for plant growth: CI < 40%, AFP = 7–50%, BD < 1.7 g cm^−3^, WHC = 20–65%, PAW > 20%, pH = 4.5–8.5, and EC < 2.2 dS m^−1^. Different letters denote significant differences among property means across pine bark rates within each mineral component (for all *p* < 0.001, except WHC *p* = 0.002, and no analysis for PAW). CI was measured on 3 pine bark rates (0, 10, and 50%). PAW was measured on 2 pine bark rates (10 and 50%).

**Figure 2:**
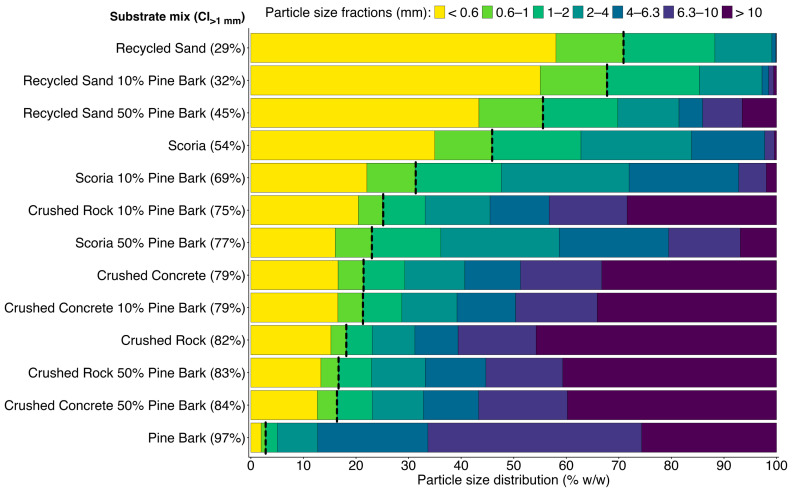
Particle size distribution of the four mineral components (recycled sand, scoria, crushed concrete, and crushed rock), pine bark, and the eight substrate mixes (10% and 50% pine bark within each mineral component) from 2 L samples. The X-axis represents % weight/weight of particles in each size fraction (coloured bars; <0.6 mm, 0.6–1 mm, 1–2 mm, 2–4 mm, 4–6.3 mm, 6.3–10 mm, and >10 mm). The Y-axis represents substrate mix or component with coarseness index (CI_>1 mm_); the cumulative percent of particles > 1 mm. Vertical black dashed lines indicate a 1 mm threshold used for CI.

Coarseness index (CI) exceeded the acceptable threshold for plant growth in pine bark, all mineral components, and substrate mixes, except for recycled sand with no pine bark and recycled sand with 10% pine bark (Figure 1i–iv). The interaction between pine bark addition rate and mineral component significantly affected CI (χ^2^_(11)_ = 994.0, *p* < 0.001). Pine bark addition significantly increased the CI of all mineral components, except crushed rock. For recycled sand, the addition of 50% pine bark increased CI beyond the acceptable threshold for plant growth (45%, Figure 1i). Particle size distributions show a clear grouping of the substrate components and mixes into three statistically distinct categories (Figure 2): “dominantly fine” (CI < 40%), comprising recycled sand with no pine bark and recycled sand with 10% pine bark; “intermediate” (CI 45–55%), comprising scoria with no pine bark and recycled sand with 50% pine bark; and “dominantly coarse” (CI > 55%), comprising all other substrate components and substrate mixes.

Mineral components scoria and crushed rock had air-filled porosity (AFP) within the acceptable range for plant growth, while the AFP of recycled sand and crushed concrete was below the threshold (Figure 1v–viii). A significant interaction between pine bark addition and mineral component affected AFP (χ^2^_(9)_ = 132.2, *p* <0.001), as shown by the variable response of AFP to increasing pine bark addition across substrate mixes. Recycled sand required the highest pine bark addition rate (50%) to achieve acceptable AFP, whereas a 10% addition was sufficient to bring crushed concrete into the acceptable range (>7%, Figure 1v,vii).

The dry bulk density (BD) of all mineral components was within the threshold for unrestricted root growth, although crushed rock was near the upper limit (1.7 g cm^−3^; Figure 1ix–xii). Increasing pine bark addition rates generally reduced BD across all mineral components, with the 50% rate producing the lowest BD values and bringing crushed rock closer in line with the other substrate mixes (Figure 1ix–xii). A significant interaction between pine bark addition rate and mineral component affected the BD of the substrate mixes (χ^2^_(9)_ = 66.4, *p* < 0.001).

All mineral components had a water-holding capacity (WHC) within the acceptable range for plant growth, although crushed rock was at the lower limit (20%, Figure 1xiii–xvi). A significant interaction between pine bark addition rate and mineral component affected WHC (χ^2^_(9)_ = 160.8, *p* < 0.001). In scoria and crushed concrete, increasing rates of pine bark addition significantly lowered WHC compared to 0% pine bark, though values remained within the acceptable range. In contrast, rates of 20% and 50% pine bark addition significantly increased WHC in crushed rock, bringing it further into the acceptable range (23% and 26%, respectively).

Plant available water (PAW) was measured only in substrate mixes with 10% and 50% pine bark addition. Adding 50% pine bark had no effect on PAW in recycled sand and scoria, increased PAW in crushed concrete, and decreased it in crushed rock (Figure 1xvii–xx). Only the recycled sand mixes had acceptable PAW for plant growth (22% and 23% *w*/*w*). All other substrate mixes had unacceptable PAW, with the lowest values observed in crushed concrete with 10% pine bark addition (5% *w*/*w*) and in both crushed rock mixes (5% and 2% *w*/*w*). Notably, 50% pine bark addition improved PAW in crushed concrete by 7% *w*/*w*, making it equivalent to both scoria mixes (12% *w*/*w*). Though not formally measured, we observed that mineral components had drastically different water retention and drainage times after irrigation, regardless of pine bark addition rates. Recycled sand drained much more slowly than the coarser components, especially crushed rock, which drained rapidly.

All mineral components were very strongly or hyper-alkaline (pH 9.2–12.3) and unsuitable for plant growth (acceptable threshold: pH < 8.5; Figure 1xxi–xxiv), but crushed concrete was the most alkaline (pH 12.3). A significant interaction between pine bark addition rate and mineral component affected pH (χ^2^_(9)_ = 171.9 and *p* < 0.001). Except for crushed concrete, adding 50% pine bark reduced pH to acceptable levels across all other mineral components, with reductions most pronounced in scoria, followed by crushed rock and recycled sand (Figure 1xxi–xxiv).

Electrical conductivity (EC) followed a similar pattern to alkalinity, with crushed concrete being the most saline (1.1 dS m^−1^, *p* < 0.001). However, all mineral components and substrate mixes had EC in the acceptable range for plant growth (<2.2 dS m^−1^; Figure 1xxv–xxviii). In crushed concrete, 50% pine bark addition significantly lowered EC, aligning it with recycled sand with no pine bark (0.4 dS m^−1^, *p* < 0.001).

### 2.2. Plant Biomass and Allocation in the Container Experiment

**Figure 3:**
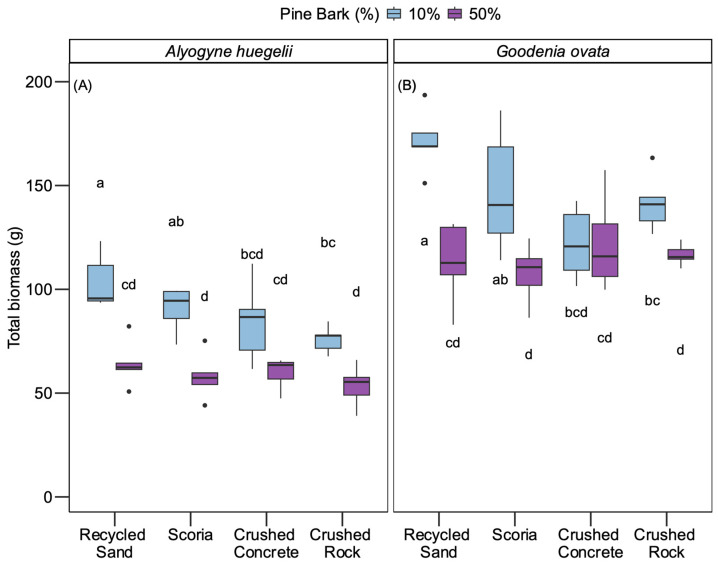
Effect of substrate mix (pine bark rate x mineral component) on total biomass (g) for (**A**) *Alyogyne huegelii* and (**B**) *Goodenia ovata*. The X-axis represents mineral component (recycled sand, scoria, crushed concrete, and crushed rock). Colours indicate pine bark rate (10% = blue, 50% = purple). Black dots indicate outlier values. Different letters above or below boxes denote significant differences among mean total biomass across the interaction of pine bark addition rate and mineral component within each species.

There was no plant mortality in the container experiment. Shoot and root biomass were highly correlated with total biomass (R = 0.97 and 0.93, *p* < 0.001); therefore, only total biomass results are presented. Both species shared similar patterns of total biomass response across substrate mixes (Figure 3). A significant interaction between pine bark addition rate and mineral component affected total biomass (χ^2^_(3)_ = 15.1, *p* = 0.002). In most substrate mixes, 50% pine bark addition resulted in lower total biomass compared to 10% addition, with the largest decrease observed in scoria and crushed rock substrates. However, in crushed concrete, the negative effect of 50% pine bark was less pronounced, with no significant difference in mean total biomass between 10% and 50% pine bark mixes (*p* = 0.2; Figure 3).

Biomass allocation—root mass fraction (RMF) and leaf mass fraction (LMF)—was not significantly affected by pine bark addition rate, mineral component, or their interaction (*p* > 0.05, Table S1).

Overall, *Goodenia ovata* was the more vigorous species, exhibiting significantly greater total, shoot, and root biomass, and LMF than *Alyogyne huegelii* when comparing within pine bark rates (all *p* < 0.001; Table S2). However, *Alyogyne huegelii* had significantly greater RMF than *Goodenia ovata* (*p* < 0.005; Table S1).

### 2.3. Relationships Between Substrate Physical and Hydrological Properties and Plant Biomass

**Figure 4:**
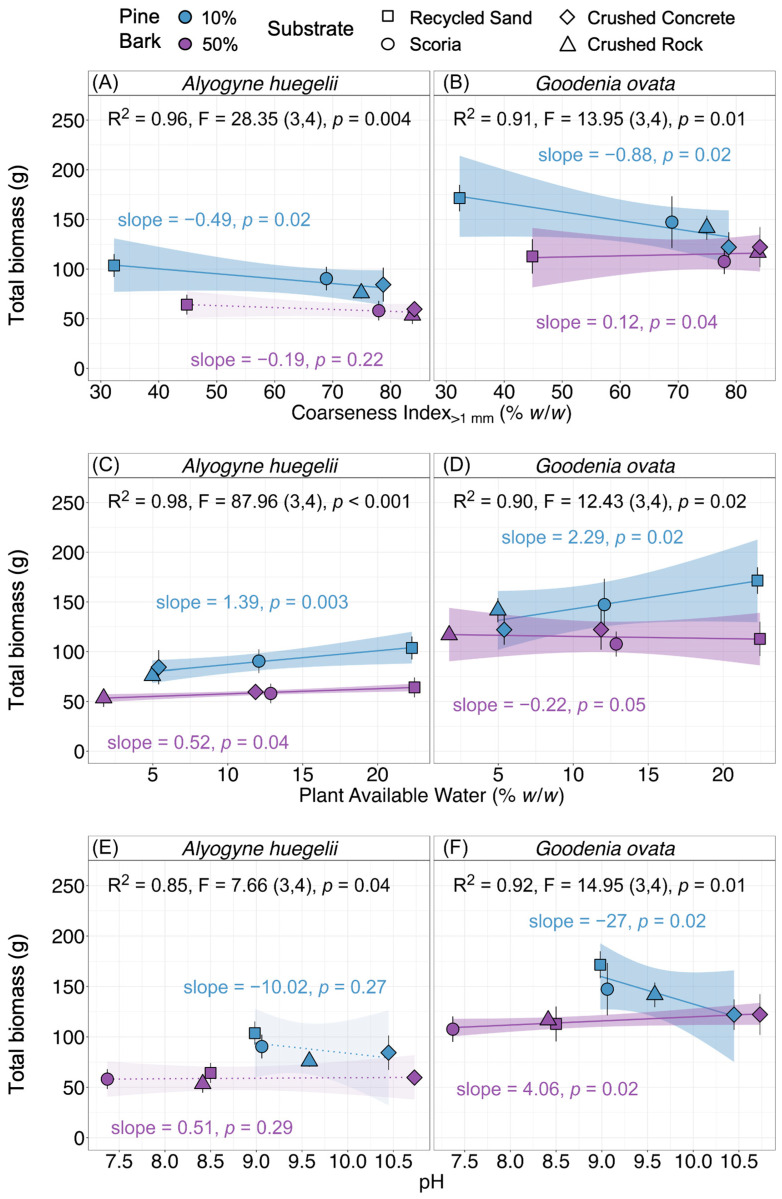
Relationships of mean total biomass (g), against (**A**,**B**) coarseness index_>1 mm_ (% *w*/*w*), (**C**,**D**) plant available water (% *w*/*w*), and (**E**,**F**) pH. Columns indicate species: *Alyogyne huegelii* (**A**,**C**,**E**) and *Goodenia ovata* (**B**,**D**,**F**). Colours indicate pine bark volume (10% = blue, 50% = purple). Symbols represent mineral components (recycled sand = squares, scoria = circles, crushed concrete = diamonds, and crushed rock = triangles). Linear relationship significance is represented by lines (solid = significant and dotted = insignificant), with shading representing standard error. Property–pine bark interaction is summarised as slope and *p*-value (*p*). Linear model fit for each property within each species is presented as model coefficient (R^2^), F-statistic (degrees of freedom), and *p*-value (*p*).

We assessed relationships between substrate physical (CI, AFP, BD) and hydrological (WHC, PAW) properties and total biomass. Air-filled porosity and CI were strongly positively correlated with each other (R = 0.8, *p* = 0.02) and exhibited similar slopes in their relationships with total biomass (Table S3). Therefore, only CI is presented here and in Figure 4 to represent physical properties. Coarseness index and PAW were strongly negatively correlated with each other (R = −0.86, *p* = 0.006). For *Alyogyne huegelii*, total biomass was significantly negatively related to CI in the 10% pine bark substrate mixes (*p* = 0.02; Figure 4A), but this relationship was not significant in the 50% mixes (*p* = 0.22). In contrast, total biomass showed a significant positive relationship with PAW in the 10% pine bark substrate mixes (*p* = 0.003; Figure 4C) and a marginally positive relationship in 50% mixes (*p* = 0.04). For *Goodenia ovata*, a similar pattern was observed; total biomass was significantly negatively related to CI in the 10% pine bark substrate mixes (*p* = 0.02; Figure 4B), but this relationship was marginal for 50% mixes (*p* = 0.04). Total biomass was significantly positively related to PAW in the 10% mixes (*p* = 0.02; Figure 4D), but this was marginal for 50% mixes (*p* = 0.05). No significant relationships were found between total biomass and either BD or WHC in either species, across both pine bark addition rates (*p* > 0.05; Table S3).

### 2.4. Relationships Between Substrate Chemical Properties and Total Biomass

We assessed the relationships between substrate chemical (pH and EC) properties and total biomass. For *Alyogyne huegelii*, there were no significant relationships between total biomass and pH in either the 10% or 50% pine bark substrate mixes (*p* = 0.2; Figure 4E). In *Goodenia ovata*, total biomass had a significant negative relationship with pH in the 10% pine bark substrate mixes (*p* = 0.02; Figure 4F), with a marginal effect of pH observed in the 50% mixes (*p* = 0.02). No significant relationships were found between total biomass and EC in either species (*p* > 0.05; Table S3).

## 3. Discussion

Results from this study demonstrate that pine bark addition improved the physical and chemical properties of alkaline construction wastes so that they may be used as capping substrates for naturalistic plantings. This study demonstrates that even at low rates of pine bark addition, construction wastes can be a sustainable substitute to quarried scoria in capping substrates, though high coarseness and low water retention may limit biomass growth, and hyper-alkaline crushed concrete may limit biomass in alkali-sensitive species.

### 3.1. Effect of Pine Bark Addition on Substrate Properties

Overall, greater rates of pine bark improved air-filled porosity (AFP) and dry bulk density (BD) across all mineral components. However, these benefits were consistently accompanied by increased coarseness index (CI) and often by reductions in water-holding capacity (WHC) or plant available water (PAW). These results generally support our hypothesis that greater rates of pine bark will improve physical properties, except in the case of CI. However, greater rates of pine bark had inconsistent effects on hydrological properties, so we cannot confirm that aspect of the hypothesis. In scoria and recycled sand, pine bark increased AFP and reduced BD, but also raised CI, which, in combination, may explain the lower WHC observed in scoria. However, PAW remained unchanged between 10% and 50% rates of pine bark in both substrates. In crushed concrete, greater rates of pine bark improved AFP, BD, and PAW, but also increased coarseness and reduced WHC. In crushed rock—the coarsest mineral component—pine bark improved AFP and BD but reduced both WHC and PAW.

These variable effects on hydrological properties can be attributed to the particle size distributions (summarised as CI) of both the mineral components and pine bark. The pine bark used was “dominantly coarse” (6–10 mm), likely increasing macro-porosity in already coarse substrates—scoria, crushed concrete, and crushed rock—leading to reduced water retention, expressed as either lower WHC or PAW. Previous studies have shown that pine bark’s particle size strongly influences its effect on substrate physical and hydrological properties [60,65]. Coarse particles (>6.4 mm) can increase AFP and drainage but lower water availability [66], whereas fine particles (<0.5 mm) can decrease AFP and increase WHC and PAW [60].

The trade-off between CI and water retention was most extreme in crushed rock substrates at both pine bark rates, where high CI and low PAW indicated faster drainage rates. The crushed rock particles had the most angular particles out of all the recycled mineral components (Figure S1). Interlocking of angular particles can create larger pores and atypical capillary action [22], which has been reported to increase drainage and decrease water retention in recycled substrate studies using crushed porcelain, demolition aggregate, and crushed brick [24,45,67]. However, crushed concrete substrates, which had similar CI to crushed rock but greater WHC and PAW, had more rounded particles. Concrete is more reactive to weathering than basalt rock as it has faster dissolution rates [68,69], which may produce less angular particles over a shorter time (concrete: 1–2 years vs. basalt: >10 years; [70,71].

In our study, pine bark addition increased AFP and decreased BD, which aligns with physical and hydrological property trade-offs observed in the literature. For example, Blythe and Merhaut [72] observed a negative relationship between total porosity (AFP and WHC) and density (BD) across 127 container media blends, including pine bark, peat, and sand. Similarly, Graceson [46] found that organic matter additions decreased BD and increased WHC, while AFP increased with coarse compost (10–25 mm) addition and decreased with finer compost (0–10 mm). However, unlike these studies, we did not observe a consistent relationship between BD and WHC or AFP and WHC. This may be due to our limited sample of mineral components, with two dominantly coarse materials and only one dominantly fine material, as well as one intermediate material, which limited the sample of CI to more extreme values on the scale. Future work should assess a greater range of materials sampling at regular intervals along the CI range to fully characterise the trade-offs between physical and hydrological properties of construction wastes.

Collectively, these results emphasise the importance of balancing particle size distribution when designing capping substrates. Overly coarse mixtures may compromise water retention, while blends with appropriate proportions of fine and coarse particles can support favourable physical and hydrological properties for plant growth [26,58].

All mineral components were too alkaline for plant growth, although salinity was within the acceptable range. Increasing pine bark addition reduced pH non-linearly, and only the 50% rate lowered pH into the acceptable range for recycled sand, scoria, and crushed rock. Crushed concrete remained hyper-alkaline even at the highest pine bark rate. These results generally support our second hypothesis that greater rates of pine bark addition improve chemical properties, except in the case of crushed concrete.

In contrast, previous green roof studies have reported that even low rates of organic matter can significantly reduce pH in strongly to hyper-alkaline mineral substrates [73,74]. For example, Molineux [54] found that a slightly acidic organic mix of 1:1 conifer-bark compost and medium clay soil, added at 15% and 25% *v*/*v* to alkaline “Carbon8” pellet green roof substrates, significantly reduced the pH from 11.8 to 9.1. Although not explicitly stated, the inclusion of medium clay soil likely contributed high proportions of mineral and organic fine particles, which may have enhanced the acidifying capacity of the organic matter, leading to the observed pH reduction [9,54].

As with physical properties, fine particles (<0.6 mm) strongly influence chemical properties such as pH by altering the composition of the substrate solution (also “soil solution”; [9,36]). In our study, the pine bark was moderately acidic (pH 5.8) but dominantly coarse (CI = 97%), with only 2% of particles < 0.6 mm. At lower addition rates (10% and 20% *v*/*v*), this equated to just 0.2% and 0.4% of total substrate volume composed of fine pine bark particles, making it unlikely that they could meaningfully alter the substrate solution.

It is therefore more likely that the pH reductions observed were driven by physical dilution, with coarse pine bark particles displacing alkaline mineral fines, rather than chemical acidification. This would explain why a high pine bark addition rate (50%) was required to reduce pH in the strongly alkaline substrates (recycled sand, scoria, and crushed rock), and why even at this rate, crushed concrete remained hyper-alkaline. Our findings highlight the limited acidifying effect of coarse pine bark, particularly in hyper-alkaline substrates like crushed concrete, where substantial additions were still insufficient to bring pH into the suitable range for plant growth.

Overall, these results reinforce the importance of particle size in shaping physical, hydrological, and chemical substrate properties. Achieving pH levels suitable for plant growth in alkaline mineral components may require not only organic amendments, but also adequate quantities of fine mineral or organic particles capable of chemically altering the substrate solution to reduce alkalinity.

### 3.2. Plant Response to Physical and Hydrological Properties Altered by Pine Bark Rate

Contrary to our first hypothesis, substrates with 10% pine bark addition had greater total biomass than substrates with 50% addition, except in crushed concrete. Within substrates with 10% pine bark, plant biomass was greatest in recycled sand and lowest in crushed rock and crushed concrete, whereas within substrates with 50% pine bark, plant biomass was similar among all mineral components.

We found that biomass was related to AFP, CI, and PAW, which were generally more suitable for plant growth in 10% mixes compared to 50% mixes, except for AFP. Recycled sand substrates were “dominantly fine” and had the highest PAW and lowest AFP, which, according to guidelines, would limit plant growth. However, we observed the opposite effect, like in the study by Ekşi and Rowe [45], where a green roof substrate with 3% AFP had the greatest biomass. As the recycled sand is a washed soil material screened to ≤3 mm, it likely contained silt and clay particles < 0.6 mm in size. As discussed, mineral and organic fines are known to increase WHC and PAW of substrates, while reducing AFP and increasing BD [9,36]. This increase in water retention can result in greater plant biomass yield [75].

Ekşi and Rowe [45] found expanded shale substrates retained more water after irrigation than coarser recycled substrates using foamed glass or crushed porcelain, resulting in greater total biomass of sedum and basil plants. They also found that even if the recycled substrates had similar or higher WHC to expanded shale, the combination of their greater coarseness (CI > 40%), considerably higher AFP, and rapid drainage resulted in lower water retention, which reduced plant biomass [45]. These results are reflected in our study, where crushed concrete and crushed rock 10% pine bark substrates and all 50% pine bark substrates had high CI and AFP, indicating greater drainage [45,75], which resulted in lower biomass compared to recycled sand with 10% pine bark.

Graceson [76] found similar relationships among coarseness, water retention, and biomass of sedum, where they identified WHC as an important predictor for water retention, and thus biomass growth on green roofs. Biomass was greatest in crushed tile substrates with 30% *v*/*v* of composted green waste, which had the highest proportion of fine particles (41% < 1 mm) and the greatest WHC (33%) compared to crushed brick and Lytag^®^ substrates [76]. However, when the rate of composted green waste was lowered to 20% *v*/*v*, all substrates had lower WHC and generally greater coarseness, which resulted in lower sedum biomass compared to 30% substrates, and similar biomass across all mineral components [76]. This aligns with the relationships we found among CI, PAW, and biomass, with increasing CI and lower PAW reducing biomass.

Our study demonstrates that substrate coarseness, which was influenced by mineral component and pine bark rate, is a key driver of available water and thus plant performance. The finest substrate mixes had the highest water availability and the best plant growth, which highlights the importance of supplying sufficient fine particles in capping substrates. We also found that while 50% pine bark addition did not affect plant survival, it reduced plant growth, which contrasts with previous green roof and agricultural studies that have demonstrated a strong positive effect of greater organic matter addition rates on plant biomass or yield [73,77]. In addition to the greater drainage and reduced water retention from 50% pine bark addition, it is possible that nitrogen availability was reduced due to microbial immobilisation (i.e., nitrogen drawdown; [78]) in response to the large carbon source provided by pine bark. This limitation may have added to the unfavourable physical and hydrological conditions, resulting in lower plant biomass in substrates with 50% pine bark. However, pine bark at 50% also reduced the variation in physical and hydrological properties among mineral components, which aligned more extreme crushed concrete and crushed rock with scoria and recycled sand, making them comparable candidates for capping substrates.

### 3.3. Alkaline-Sensitive Plant Response to pH Altered by Coarseness

Despite greater rates of pine bark improving chemical properties in recycled sand, scoria, and crushed rock, this did not translate to greater biomass in either species, as 50% mixes generally had worse growth. For alkaline-sensitive *Goodenia ovata*, biomass was similar between pine bark rates in crushed concrete, where the pH was extreme and unaffected by increased pine bark addition. The biomass of both species in 50% mixes seemed to be influenced more by physical properties, rather than pH.

However, in 10% mixes, there was clear evidence of an alkalinity effect on *G. ovata*, with hyper-alkaline crushed concrete resulting in the lowest biomass of all the mineral components. Poor growth of alkaline-sensitive plants in concrete-based substrates was documented in a green roof study where meadow species with a strong preference for acid soils did not survive, but species with broad pH tolerance or alkaline tolerance had high survival and abundance [47]. Despite the high alkalinity of the substrates in our study, there was no mortality of either species in any of the substrate mixes, and though the biomass of sensitive species was reduced in crushed concrete substrates, it was no different from substrates with 50% pine bark, where all mineral components had similar biomass. This is a promising outcome which shows construction wastes such as recycled sand, crushed rock, and even the hyper-alkaline crushed concrete, may be used as capping substrates in place of scoria, the current preferred mineral material for capping substrates in woody naturalistic plantings. However, construction wastes must be amended with pine bark as their properties are too extreme for plant growth.

### 3.4. Limitations

We focused on only two plant species to understand how alkalinity may affect plant growth using waste substrates. However, to assess the broader application of construction wastes as capping substrates in urban naturalistic plantings, experiments with a larger plant palette are required. In addition, container studies of monocultures are not always representative of true field conditions in the urban landscape [24]. Future work should assess these capping substrates overlying urban soil and the interacting effect of substrate depth on plant performance. Further, to comprehensively compare construction wastes to industry standards like scoria and sand, we must understand how they can function as mulch to suppress weeds. Future work should assess the viability of construction wastes functioning as mulch in comparison to sand or scoria. The results obtained in this study show that pine bark-amended construction wastes can support plant growth in containers, but similar experiments in a landscape context are required to fully validate their function as capping substrates in urban naturalistic plantings.

## 4. Materials and Methods

### 4.1. Substrate Components

Four types of mineral components were evaluated, scoria—as a reference mineral component—and three recycled waste materials: recycled sand, crushed concrete, and crushed rock. The scoria was sourced from Aerolite Quarries Pty Ltd. (Anakie, VIC, Australia) and is a quarried volcanic material, crushed and screened to ≤8 mm, including fine particles. The recycled materials were sourced from Repurpose It^®^ Pty Ltd. (Epping, VIC, Australia), a construction and demolition waste recycling company based in Melbourne, Australia. The crushed concrete and crushed rock materials are crushed and screened to ≤20 mm minus, and can have variable origins, depending on supply from construction projects. The crushed concrete used in this experiment was sourced from construction and demolition mixed waste from the Greater Melbourne region and included 1.7% metal, glass, brick, ceramics, or slag. The crushed rock used in this experiment is composed of Tertiary Olivine basalt, which is naturally alkaline, excavated from the Greater Melbourne region. The recycled sand is a washed and screened soil material derived from EPA category C and D contaminated soils [79]. The recycled sand is processed through a wash plant to remove contaminants and then screened to ≤3 mm, including fines.

The organic component—aged *Pinus radiata* bark mulch (hereafter “pine bark”)—was sourced from Repurpose It^®^ Pty Ltd. (Epping, VIC, Australia) and is a screened waste material from the timber industry (6–10 mm). The pine bark had the following properties: high coarseness index (CI = 97% *w*/*w*), high air-filled porosity (AFP = 49% *v*/*v*), low dry bulk density (BD = 0.2 g cm^−3^), moderate water-holding capacity (WHC = 25% *v*/*v*), moderate acidity (pH = 5.8), and low salinity (EC = 0.2 dS m^−1^). Pine bark typically has low nitrogen content, between 0.2 and 0.76% dry mass [9,80]. Figure S1 shows the appearance of each substrate component.

### 4.2. Physical and Chemical Properties of Substrate Components and Mixes

Substrate mineral and organic components and substrate mixes were tested for physical, hydrological, and chemical properties. The four mineral components were mixed with three rates of pine bark (10%, 20%, and 50% *v*/*v*) to create 12 different substrate mixes for testing physical (AFP and BD), hydrological (WHC), and chemical properties (pH and EC). Particle size distribution, summarised as coarseness index (CI), was tested on the individual substrate components and on substrate mixes with 10% or 50% *v*/*v* pine bark addition. Plant available water was evaluated on mixes with 10% or 50% *v*/*v* pine bark addition. Table 1 describes the acceptable ranges for each substrate property.

Physical properties and WHC (all water held in substrate below −10 kPa) were measured on three replicates of each substrate component and substrate mix. Air-filled porosity, WHC, and BD were measured using methods described in the Australian Standard for potting mixes (AS 3743-2003, [31]). In summary, using a special apparatus (described in Conn [33]), approximately 720 mL of each substrate mix was immersed in water to soak for 30 min and then drained for 5 min, followed by two more soak (10 min)-and-drain (5 min) cycles. Then, the top part of the apparatus (as described in Conn [33]) was removed, and excess substrate was scraped flush with the top of the apparatus base. The apparatus was then immersed a final time for 2 min to achieve full saturation, then drained into a measuring container for 30 min. Before the last drain period, drainage holes at the bottom of the apparatus were sealed before the samples were removed from the water to ensure all water held within the apparatus was kept and released into the measuring container. The volume of the final drained water (V_water_) was measured in mL and used with the substrate volume (V_sub_) to calculate AFP:

(1) $$AFP\;(\%\;v/v)=\left(\frac{V_{water}}{V_{sub}}\right)\times 100$$

The ~720 mL substrate was then oven-dried (105 °C for 5 days) and weighed. The substrate oven-dry weight (W_dry_ in g), saturated weight after 30 min of drainage (W_sat_ in g), and its volume were used to calculate WHC and dry BD.

(2) $$WHC\;(\%\;w/w)=\left(\frac{W_{sat}-W_{dry}}{V_{sub}}\right)\times 100$$

(3) $${BD}_{dry}(g\;{cm}^{3})=\frac{W_{dry}}{V_{sub}}$$

Particle size distribution was measured following the methods described in Cao [32] and Werdin [58]. Substrates were oven-dried at 105 °C to achieve constant weight, and two replicates of 1 L were passed through a nest of sieves in descending order from 10 mm, 6.3 mm, 4 mm, 2 mm, 1 mm to 0.6 mm aperture. The coarseness index [28] was calculated as the weight of all particles > 1 mm (W_>1 mm_ in g) divided by the total sample weight (W_Total_ in g):

(4) $${CI}_{>1mm}\%(w/w)=\left(\frac{W_{>1\;mm}}{W_{Total}}\right)\times 100$$

Plant available water was calculated as moisture content (%) measured at container capacity (−10 kPa) minus moisture content at the permanent wilting point (−1500 kPa). To measure moisture content at container capacity, we adapted the methods of Nelson [81] for use with coarse substrates > 2 mm in particle size. Duplicate samples were placed into 100 mm diameter funnels with Whatman no. 1 filter paper and saturated with deionised water, and then left to drain for 24 h. Wet substrate (W_wet_ in g) samples were weighed, then oven-dried (105 °C for 4 days) and reweighed (W_dry_ in g) to determine moisture content at container capacity as percentage weight (% *w*/*w*):

(5) $$Moisture\;content\;\%(w/w)=\left(W_{wet}-W_{dry}\right)\times 100$$

To measure moisture content at permanent wilting point, we adapted the pressure plate method described in Yeates [82] for use with coarse substrates > 2 mm in particle size. Approximately 70 mL of each substrate mix was placed into 50 mm diameter × 30 mm deep metal rings on a ceramic pressure plate and then saturated with deionised water overnight. The ceramic pressure plate with the saturated samples was then placed into the pressure plate apparatus (Soilmoisture Equipment Corp., CA, USA), sealed, and the upper chamber was slowly brought up to 1500 kPa, and the sample moisture content was left to reach equilibrium over seven days. The wet substrate samples were weighed after removal from the pressure plate apparatus, then oven-dried (105 °C for 5 days) and reweighed to determine moisture content at permanent wilting point as % *w*/*w* using Equation (5).

Chemical properties were measured on three replicates of each substrate component and substrate mix. We measured EC and pH in a 1:5 substrate to water solution, according to the Australian standard for soils for landscaping and garden use (Appendix D; AS 4419-2018, [38]) using a lab-bench pH and conductivity meter (labCHEM, TPS International Pty Ltd., Brisbane, QLD, Australia). To account for the larger particle sizes of the substrate components, we adjusted the method to use 80 g of air-dried substrate and 400 mL of deionised water in a 500 mL bottle for each sample. Substrate–water solutions were shaken for one hour in an end-to-end shaker, and pH was measured immediately. The solution was then left to settle overnight and then filtered using Whatman No. 1 filter papers before measuring EC.

### 4.3. Plant Growth Experiment

We evaluated the plant response of two species in a container experiment undertaken at the Burnley Campus, University of Melbourne, Australia (latitude 37°47′ S; longitude 144°58′ E). Eight substrate mixes, composed of the four mineral components (recycled sand, scoria, crushed concrete, crushed rock) and pine bark at two rates of addition (10% and 50% *v*/*v*), were evaluated. Substrate components were mixed for two minutes in a clean 99 L concrete mixer (Lightburn & Co., Ltd., Adelaide, SA, Australia) to create a total of 126 L of each substrate mix for the experiment. After mixing, substrates were dumped on a concrete pad and turned manually with a shovel for one minute to incorporate coarse particles. We filled 10 replicates of 10.5 L “Citrus” containers (d 250 mm × h 275 mm, P250C, Garden City Plastics Pty Ltd., Dandenong South, VIC, Australia) with nine litres of each substrate mix for a total of 80 containers. Substrate was lightly shaken to level but not compact it in each container.

**Figure 5:**
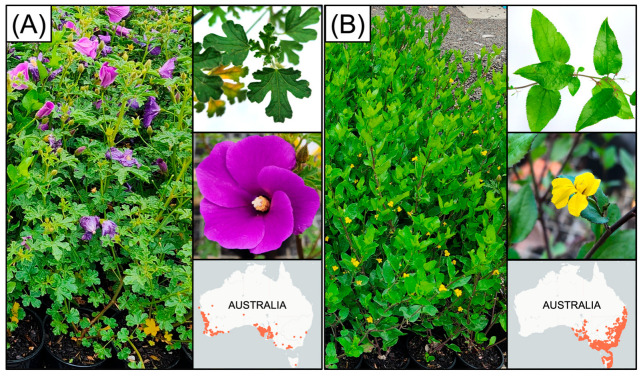
The two woody plant species used in the experiment are (**A**) *Alyogyne huegelii* and (**B**) *Goodenia ovata*. Photos show the whole plant, leaf, and flower morphology. Species occurrences in Australia are displayed in the bottom panel for each species, adapted from Atlas of Living Australia [83,84], red dots indicate occurrences.

We selected two vigorous fast-growing woody plant species that have been shown to perform well in previous installations of woody naturalistic plantings using a scoria capping substrate [62]: *Alyogyne huegelii* and *Goodenia ovata*. *A. huegelii* is a medium-sized ornamental shrub up to 2.5 m high with dull green, hairy, deeply three- to five-lobed leaves and large, lilac or mauve flowers [83] (Figure 5A). *G. ovata* is a small–medium-sized ornamental shrub up to 1 m high with glossy green oval leaves that have toothed margins, and small bright yellow fan-like flowers [84] (Figure 5B). These species have contrasting tolerance of alkalinity; *A. huegelii* tolerates acidic to alkaline conditions [63], whereas *G. ovata* tolerates acidic to neutral conditions [64].

Mature plants were purchased in 14 cm pots from a wholesale nursery (Plantmark Pty Ltd., Melbourne, VIC, Australia). Planting into containers filled with substrate mixes was completed on 6 March 2023 (late summer), with each plant receiving a surface application of controlled-release fertiliser (2.7 g per container of Osmocote^®^ Pro Low P, Scotts Australia Pty. Ltd., Sydney, NSW, Australia; NPK of 14:1.3:14.9), equivalent to 0.38 g nitrogen per plant. One week after planting, all plants were pruned to 40 cm above the substrate surface to promote multi-stemmed growth. All plants also received additional liquid fertiliser applications (1 L per container) of Peters Professional^®^ All Rounder (NPK of 20:8.7:16.6, rate: 0.5 g L^−1^), equivalent to 0.1 g nitrogen per plant on four occasions; once in May and October, and twice in November 2023. Plants were irrigated two to four times daily with overhead rotary sprinklers (12 L min^−1^) except for three months over the winter (May–August 2023). Additional hand-watering (approximately 1 L per container) occurred once (18 March 2023) when the maximum daily temperature exceeded 35 °C. Although soil moisture was not directly monitored, it is unlikely that containers remained at saturated WHC following irrigation events, given the inherent water loss through large container drainage holes.

### 4.4. Plant Biomass and Allocation

Plants were destructively harvested after 10 months of growth (March 2023 to January 2024) to measure shoot, leaf, and root biomass. Above-ground biomass was removed and separated into leaves and stems. Roots were removed from the containers and carefully washed to remove the substrate mix. Stems, leaves, and roots were oven-dried at 60 °C until a constant dry weight was achieved. Dry mass was used to calculate total biomass (g), and plant allocation metrics; root mass fraction (g g^−1^; RMF), and leaf mass fraction (g g^−1^; LMF).

### 4.5. Statistical Analyses

All statistical analyses were performed in R version 4.1.2 [85]. All response variables (substrate properties and plant biomass or allocation) were tested for normality using the Shapiro–Wilk test.

To test whether greater rates of pine bark addition improved physical, hydrological, and chemical properties and resulted in greater plant growth, we used linear regression to analyse the normally distributed response variables. These included substrate properties (AFP, BD, pH, and WHC) and plant traits (mean total, shoot, and root biomass; RMF; and LMF). Response variables were analysed against treatments of pine bark addition rate, mineral component, and their interaction (substrate mix). To test the significance of treatments, we used two-way ANOVA (type III) from the “car package” [86]. Where significant effects were found, the “emmeans” package [87] was used to determine estimates of the group means for each response variable and differences between group means were tested with post hoc pairwise comparisons using Tukey’s HSD method at a significance level of 0.05. Mean pairwise comparisons were performed (1) across pine bark rates (0, 10, 20, and 50% *v*/*v*) within each mineral component, and (2) among substrate mixes (interaction term) for each response variable. Analyses of plant response were conducted separately for each species.

For right-skewed EC, transformations could not achieve a normal distribution, so generalised linear regression using the Gamma family with a log link was used to analyse the response against treatments of pine bark addition rate, mineral component, and substrate mix. For the left-skewed CI, values were bounded (0–100%) and so were rescaled to a proportion (0–1) and then analysed with beta regression using the “betareg” package [88]. Post hoc pairwise comparisons of estimated means among pine bark addition rates were performed within each mineral component and across substrate mixes, with Bonferroni-adjusted *p*-values to control for multiple comparisons of the four pine bark addition rates and eight substrate mixes.

Plant available water (PAW) was excluded as a response variable due to limited replication (*n* = 1 per substrate mix) and is summarised descriptively.

To test whether improved substrate properties led to greater plant growth, we used linear regression models with plant biomass and allocation as response variables and substrate properties as predictors, including interaction terms with pine bark rate. Analyses were conducted separately for each species. We report slope estimates, model fit (R^2^, F-statistic, *p*-value), and significant interactions. Degrees of freedom for the F-statistic are reported as “F(df1, df2)”. Collinearity among substrate properties was tested using Pearson’s correlation from the “psych” package [89], with a significance threshold < 0.05. The correlation coefficient (R) among properties is reported in text as (R, *p*-value).

To test whether reduced alkalinity led to better growth of the alkaline-sensitive species (*G. ovata*) compared to the tolerant species (*A. huegelii*), we used linear regression models with plant traits as response variables and species as the predictor. Where significant effects were found, differences between group means were tested with post hoc pairwise comparisons using Tukey’s HSD method at a significance level of *p*-value < 0.05.

Significant pairwise or multiple comparison differences were visualised using compact letter displays from the “multcomp” package [90]; groups sharing the same letter are not statistically different. All figures were generated using the ggplot2 package [91]. Data shown in figures and tables are on the original (non-transformed) scale.

## 5. Conclusions

The three recycled mineral components and scoria did not have acceptable properties for plant growth without pine bark addition. While greater pine bark rates generally improved the physical properties of the mineral substrates more than lower rates, this did not translate to greater plant growth. In the nursery experiment, *Goodenia ovata* had greater biomass than *Alyogyne huegelii*, but its growth was reduced by high alkalinity in 10% pine bark and high coarseness in 50% pine bark substrates. Across mineral components, 10% pine bark mixes had greater biomass than 50% pine bark mixes for both species, and recycled sand with 10% pine bark had the greatest biomass out of the construction wastes. However, biomass was similar in all substrates with 50% pine bark addition. We identified that dominantly fine substrates that had high PAW and lower alkalinity, such as recycled sand with 10% pine bark, were the best candidates for increasing plant biomass. This emphasises the importance of balancing particle size so that there are enough mineral and organic fine particles to increase water retention and lower pH. We have identified recycled sand, crushed concrete, and crushed rock as three new recycled materials that can be used with pine bark as capping substrates for urban woody naturalistic plantings. Replacing scoria with these recycled materials could enhance the sustainability of capping substrates for naturalistic plantings, while enhancing the biodiversity and amenity of informal green spaces.

## Figures and Tables

**Table 1:** Key substrate properties and their recommended ranges for healthy plant growth in urban landscape plantings based on guidelines for container media, green roof substrates, and landscape soils.

Property	Unit	Definition	Acceptable Range	References
Air-filled porosity (AFP)	% *v*/*v*	The percentage of substrate that is composed of air-filled pores	7–50% depending on plant species	[29,30,31,34,35]
Dry bulk density (BD)	g cm^−3^	Mass in grams of a dried cubic centimetre of substrate	≤1.7 g cm^−3^	[29,32,36,38]
Coarseness index (CI)	% *w*/*w*	Summary of particle size distribution: cumulative percentage by weight of particle sizes > 1 mm	<40%	[28]
Water-holding capacity (WHC)	% *v*/*v*	The total amount of water able to be held by a substrate after gravity drainage (~−10 kPa)	20–65%	[30,31,32,33,35]
Plant available water (PAW)	% *w*/*w*	The amount of water available to a plant between field capacity (−10 kPa) and permanent wilting point (−1500 kPa)	>20% depending on plant species water use	[29]
pH	unitless	The concentration of hydrogen ions (H^+^) in a medium. Indicates if the substrate is acidic, neutral, or alkaline	4.5–8.5	[29,30,32,36,39]
Electrical conductivity (EC)	dS m^−1^	The sum of all salt ions in a solution which indicates the salinity of the substrate	≤2.2 dS m^−1^	[29,30,37]

## Data Availability

The original data presented in the study are openly available in FigShare at https://doi.org/10.26188/30918680.

## Supplementary Materials

The following supporting information can be downloaded at: https://www.mdpi.com/article/10.3390/plants15030403/s1, Figure S1: Photographs illustrating the shape and size of particles in the five substrate components. Table S1: ANOVA output of pine bark addition rate and mineral component effects on plant response variables. Table S2: Pearson correlation of substrate physical, hydrological, and chemical properties. Table S3: Linear regression model output of substrate properties and pine bark addition rate effects on total plant biomass, per species.

## Abbreviations

The following abbreviations are used in this manuscript:

AFP	Air-filled porosity
AS	Australian standard
BD	Dry bulk density
CI	Coarseness index
EC	Electrical conductivity
EPA	Environmental protection agency
LMF	Leaf mass fraction
PAW	Plant available water
RMF	Root mass fraction
WHC	Water-holding capacity

## References

[1] Babington A., Hughes M., Farrell C., Chambers J., Standish R.J. (2023). Preference for Multi-Layered, Flowering, Woody Streetscape Plantings in a Mediterranean-Type Climate. Urban For. Urban Green..

[2] Hitchmough J., Wagner M., Ahmad H. (2017). Extended Flowering and High Weed Resistance within Two Layer Designed Perennial “Prairie-Meadow” Vegetation. Urban For. Urban Green..

[3] Bretzel F., Vannucchi F., Pezzarossa B., Paraskevopoulou A., Romano D. (2024). Establishing Wildflower Meadows in Anthropogenic Landscapes. Front. Hortic..

[4] Horsfall K., Livesley S.J., Delpratt J., Hirst M.J., Williams N.S.G. (2024). Interacting Effects of Sand, Slugs and Jute Drive Community Composition in Direct-seeded Urban Wildflower Meadows. J. Appl. Ecol..

[5] Craul P.J. (1991). Urban Soil: Problems and Promise. Arnoldia.

[6] Fischer L.K., von der Lippe M., Rillig M.C., Kowarik I. (2013). Creating Novel Urban Grasslands by Reintroducing Native Species in Wasteland Vegetation. Biol. Conserv..

[7] Sloan J.J., Ampim P.A.Y., Basta N.T., Scott R. (2012). Addressing the Need for Soil Blends and Amendments for the Highly Modified Urban Landscape. SSSAJ.

[8] Horsfall K., Williams N.S.G., Michael R.N., Livesley S.J. (2024). Rapid Root Development in Clay Subsoils Enhances the Early Growth of Native Grassland Species. Plant Soil.

[9] Jim C.Y. (1996). Edaphic Properties and Horticultural Applications of Some Common Growing Media. Commun. Soil Sci. Plant Anal..

[10] Rojas J.A., Dhar A., Naeth M.A. (2022). Urban Green Spaces Restoration Using Native Forbs, Site Preparation and Soil Amendments—A Case Study. Land.

[11] Bretzel F., Vannucchi F., Pini R., Scatena M., Marradi A., Cinelli F. (2020). Use of Coarse Substrate to Increase the Rate of Water Infiltration and the Bearing Capacity in Tree Plantings. Ecol. Eng..

[12] Somerville P.D., May P.B., Livesley S.J. (2018). Effects of Deep Tillage and Municipal Green Waste Compost Amendments on Soil Properties and Tree Growth in Compacted Urban Soils. J. Environ. Manag..

[13] Schwartz S.S., Smith B. (2016). Restoring Hydrologic Function in Urban Landscapes with Suburban Subsoiling. J. Hydrol..

[14] Somerville P.D., Farrell C., May P.B., Livesley S.J. (2020). Biochar and Compost Equally Improve Urban Soil Physical and Biological Properties and Tree Growth, with No Added Benefit in Combination. Sci. Total Environ..

[15] Valkó O., Kelemen A., Kiss O., Bátori Z., Kiss R., Deák B. (2024). Grassland Restoration on Linear Landscape Elements—Comparing the Effects of Topsoil Removal and Topsoil Transfer. BMC Ecol. Evol..

[16] Gibson-Roy P. (2022). Australian Grassy Community Restoration: Recognizing What Is Achievable and Charting a Way Forward. Ecol. Manag. Restor..

[17] Morris E.C., Gibson-Roy P. (2017). Comparison of Biomass Removal, Nutrient Manipulation and Native Seed Addition to Restore the Ground Layer of a Degraded Grassy Woodland. Aust. J. Bot..

[18] Hölzel N., Otte A. (2003). Restoration of a Species-rich Flood Meadow by Topsoil Removal and Diaspore Transfer with Plant Material. Appl. Veg. Sci..

[19] Jaunatre R., Buisson E., Dutoit T. (2013). Topsoil Removal Improves Various Restoration Treatments of a Mediterranean Steppe (La Crau, Southeast France). Appl. Veg. Sci..

[20] Resch M.C., Schütz M., Graf U., Wagenaar R., van der Putten W.H., Risch A.C. (2019). Does Topsoil Removal in Grassland Restoration Benefit Both Soil Nematode and Plant Communities?. J. Appl. Ecol..

[21] Buisson E., Holl K.D., Anderson S., Corcket E., Hayes G.F., Torre F., Peteers A., Dutoit T. (2006). Effect of Seed Source, Topsoil Removal, and Plant Neighbor Removal on Restoring California Coastal Prairies. Restor. Ecol..

[22] Hitchmough J., Kendle T., Paraskevopoulou A.T. (2001). Seedling Emergence, Survival and Initial Growth of Forbs and Grasses Native to Britain and Central/Southern Europe in Low Productivity Urban “Waste” Substrates. Urban Ecosyst..

[23] Egendorf S.P., Cheng Z., Deeb M., Flores V., Paltseva A., Walsh D., Groffman P., Mielke H.W. (2018). Constructed Soils for Mitigating Lead (Pb) Exposure and Promoting Urban Community Gardening: The New York City Clean Soil Bank Pilot Study. Landsc. Urban Plan..

[24] Hitchmough J.D., Kendle A.D., Paraskevopoulou A.T. (2003). Emergence, Survival and Initial Growth of North American Prairie Forbs and British Meadow Forbs and Grasses in Low-Productivity Urban “Waste” Soils. J. Hortic. Sci..

[25] Barrett G.E., Alexander P.D., Robinson J.S., Bragg N.C. (2016). Achieving Environmentally Sustainable Growing Media for Soilless Plant Cultivation Systems—A Review. Sci. Hortic..

[26] Jim C.Y. (1998). Urban Soil Characteristics and Limitations for Landscape Planting in Hong Kong. Landsc. Urban Plan..

[27] Séré G., Schwartz C., Ouvrard S., Sauvage C., Renat J.-C., Morel J.L. (2008). Soil Construction: A Step for Ecological Reclamation of Derelict Lands. J. Soils Sediments.

[28] Abad M., Fornes F., Carrión C., Noguera V., Noguera P., Maquieira Á., Puchades R. (2005). Physical Properties of Various Coconut Coir Dusts Compared to Peat. HortScience.

[29] Handreck K.A., Black N.D. (2010). Growing Media for Ornamental Plants and Turf.

[30] Mathers H.M., Lowe S.B., Scagel C., Struve D.K., Case L.T. (2007). Abiotic Factors Influencing Root Growth of Woody Nursery Plants in Containers. HortTech.

[31] (2003). Australian Standard for Potting Mixes.

[32] Cao C.T.N., Farrell C., Kristiansen P.E., Rayner J.P. (2014). Biochar Makes Green Roof Substrates Lighter and Improves Water Supply to Plants. Ecol. Eng..

[33] Conn R., Werdin J., Rayner J.P., Farrell C. (2020). Green Roof Substrate Physical Properties Differ between Standard Laboratory Tests Due to Differences in Compaction. J. Environ. Manag..

[34] Farrell C., Cao C.T.N., Ang X.Q., Rayner J.P. (2016). Use of Water-Retention Additives to Improve Performance of Green Roof Substrates. Acta Hortic..

[35] Landscape Development and Landscaping Research Society e.V. (FLL) (2018). Guidelines for the Planning, Construction and Maintenance of Green-Roofs.

[36] Craul P.J. (1994). The Nature of Urban Soils: Their Problems and Future. Arboric. J..

[37] Hazelton P., Murphy B. (2016). Interpreting Soil Test Results.

[38] (2018). Australian Standard for Soils for Landscaping and Garden Use.

[39] Ampim P.A.Y., Sloan J.J., Cabrera R.I., Harp D.A., Jaber F.H. (2010). Green Roof Growing Substrates: Types, Ingredients, Composition and Properties. J. Environ. Hortic..

[40] Kazemi F., Mohorko R. (2017). Review on the Roles and Effects of Growing Media on Plant Performance in Green Roofs in World Climates. Urban For. Urban Green..

[41] United Nations DESA (2015). Transforming Our World: The 2030 Agenda for Sustainable Development.

[42] Jiang N., Zou W., Lu Y., Liao Z., Wu L. (2024). Using Recycled Construction Waste Materials with Varying Components and Particle Sizes for Extensive Green Roof Substrates: Assessment of Its Effects on Vegetation Development. Sustainability.

[43] Kader S., Gratchev I., Michael R.N. (2024). Recycled Waste Substrates: A Systematic Review. Sci. Total Environ..

[44] Bates A.J., Sadler J.P., Greswell R.B., Mackay R. (2015). Effects of Recycled Aggregate Growth Substrate on Green Roof Vegetation Development: A Six Year Experiment. Landsc. Urban Plan..

[45] Eksi M., Rowe D.B. (2016). Green Roof Substrates: Effect of Recycled Crushed Porcelain and Foamed Glass on Plant Growth and Water Retention. Urban For. Urban Green..

[46] Graceson A., Hare M., Hall N., Monaghan J. (2014). Use of Inorganic Substrates and Composted Green Waste in Growing Media for Green Roofs. Biosyst. Eng..

[47] Jauni M., Kuoppamäki K., Hagner M., Prass M., Suonio T., Fransson A.-M., Lehvävirta S. (2020). Alkaline Habitat for Vegetated Roofs? Ecosystem Dynamics in a Vegetated Roof with Crushed Concrete-Based Substrate. Ecol. Eng..

[48] Barredo O., Vilela J., Garbisu C., Besga G., Alkorta I., Epelde L. (2020). Technosols Made from Urban and Industrial Wastes Are a Good Option for the Reclamation of Abandoned City Plots. Geoderma.

[49] Cannavo P., Guénon R., Galopin G., Vidal-Beaudet L. (2018). Technosols Made with Various Urban Wastes Showed Contrasted Performance for Tree Development during a 3-Year Experiment. Environ. Earth Sci..

[50] Rokia S., Séré G., Schwartz C., Deeb M., Fournier F., Nehls T., Damas O., Vidal-Beaudet L. (2014). Modelling Agronomic Properties of Technosols Constructed with Urban Wastes. Waste Manag..

[51] Weiler J., Firpo B.A., Schneider I.A.H. (2020). Technosol as an Integrated Management Tool for Turning Urban and Coal Mining Waste into a Resource. Miner. Eng..

[52] Pruvost C., Mathieu J., Vallet J., Dubs F., Gigon A., Lerch T., Blouin M. (2025). Technosols Made of Urban Wastes Are Suitable Habitats for Flora and Soil Macrofauna. Ecol. Eng..

[53] Mikajlo I., Pando A., Robain H., Lerch T.Z. (2024). Reusing Asphalt Millings with Excavated Materials and Compost to Construct Technosols: Effects on Soil Properties and Plant Growth. J. Soils Sediments.

[54] Molineux C.J., Fentiman C.H., Gange A.C. (2009). Characterising Alternative Recycled Waste Materials for Use as Green Roof Growing Media in the U.K. Ecol. Eng..

[55] Bretzel F., Vannucchi F., Romano D., Malorgio F., Benvenuti S., Pezzarossa B. (2016). Wildflowers: From Conserving Biodiversity to Urban Greening—A Review. Urban For. Urban Green..

[56] Graceson A., Hare M., Monaghan J., Hall N. (2013). The Water Retention Capabilities of Growing Media for Green Roofs. Ecol. Eng..

[57] Gruda N. (2019). Increasing Sustainability of Growing Media Constituents and Stand-Alone Substrates in Soilless Culture Systems. Agronomy.

[58] Werdin J., Conn R., Fletcher T.D., Rayner J.P., Williams N.S.G., Farrell C. (2021). Biochar Particle Size and Amendment Rate Are More Important for Water Retention and Weight of Green Roof Substrates than Differences in Feedstock Type. Ecol. Eng..

[59] Duryea M.L., English R.J., Hermansen L.A. (1999). A Comparison of Landscape Mulches: Chemical, Allelopathic, and Decomposition Properties. J. Arboric..

[60] Handreck K.A. (1983). Particle Size and the Physical Properties of Growing Media for Containers. Commun. Soil Sci. Plant Anal..

[61] Altland J.E., Locke J.C., Krause C.R. (2014). Influence of Pine Bark Particle Size and pH on Cation Exchange Capacity. HortTech.

[62] Farrell C., Rayner J.P., Bathgate R. (2025). Woody Meadow Guidelines: Naturalistic Plantings of Australian Woody Plants for People and Nature.

[63] Botanic Gardens of South Australia Plant Selector—*Alyogyne huegelii*. https://plantselector.botanicgardens.sa.gov.au/Plants/Details/863.

[64] Botanic Gardens of South Australia Plant Selector—*Goodenia ovata*. https://plantselector.botanicgardens.sa.gov.au/Plants/Details/565.

[65] Nkongolo N.V., Caron J. (1999). Bark Particle Sizes and the Modification of the Physical Properties of Peat Substrates. Can. J. Soil Sci..

[66] Gartner J.B., Still S.M., Klett J.E. (1974). The Use of Bark Waste as a Substrate in Horticulture. Acta Hortic..

[67] Eksi M., Sevgi O., Akburak S., Yurtseven H., Esin İ. (2020). Assessment of Recycled or Locally Available Materials as Green Roof Substrates. Ecol. Eng..

[68] del Pilar León H., Martinez S., del Mar Delgado M., Gabriel J.L., Alvarez S. (2025). Plant and Soil Responses to Concrete and Basalt Amendments Under Elevated CO_2_: Implications for Plant Growth, Enhanced Weathering and Carbon Sequestration. Agriculture.

[69] Knapp W.J., Tipper E.T. (2022). The Efficacy of Enhancing Carbonate Weathering for Carbon Dioxide Sequestration. Front. Clim..

[70] Sanger M., Natarajan B.M., Wang B., Edil T., Ginder-Vogel M. (2020). Recycled Concrete Aggregate in Base Course Applications: Review of Field and Laboratory Investigations of Leachate pH. J. Hazard. Mater..

[71] Burke T.M., Kamber B.S., Rowlings D. (2025). Microscopic Investigation of Incipient Basalt Breakdown in Soils: Implications for Selecting Products for Enhanced Rock Weathering. Front. Clim..

[72] Blythe E.K., Merhaut D.J. (2007). Grouping and Comparison of Container Substrates Based on Physical Properties Using Exploratory Multivariate Statistical Methods. HortScience.

[73] Nagase A., Dunnett N. (2011). The Relationship between Percentage of Organic Matter in Substrate and Plant Growth in Extensive Green Roofs. Landsc. Urban Plan..

[74] Xue M., Farrell C. (2020). Use of Organic Wastes to Create Lightweight Green Roof Substrates with Increased Plant-Available Water. Urban For. Urban Green..

[75] Ekşi M., Rowe D.B. (2019). Effect of Substrate Depth and Type on Plant Growth for Extensive Green Roofs in a Mediterranean Climate. J. Green. Build..

[76] Graceson A., Monaghan J., Hall N., Hare M. (2014). Plant Growth Responses to Different Growing Media for Green Roofs. Ecol. Eng..

[77] Barzegar A.R., Yousefi A., Daryashenas A. (2002). The Effect of Addition of Different Amounts and Types of Organic Materials on Soil Physical Properties and Yield of Wheat. Plant Soil.

[78] Abbruzzini T.F., Mora L., Prado B. (2021). Evaluation of Technosols Constructed with Construction and Excavation Debris for Greenhouse Production of Ornamental Plants. J. Soils Sediments.

[79] Environment Protection Authority Victoria (2021). Waste Disposal Categories—Characteristics and Thresholds.

[80] Webber B., Madgwick H.A.I. (1983). Biomass and Nutrient Content of a 29-Year-Old. N. Z. J. For. Sci..

[81] Nelson J.T., Adjuik T.A., Moore E.B., VanLoocke A.D., Ramirez Reyes A., McDaniel M.D. (2023). A Simple, Affordable, Do-It-Yourself Method for Measuring Soil Maximum Water Holding Capacity. Commun. Soil Sci. Plant Anal..

[82] Yeates G.W., Dando J.L., Shepherd T.G. (2002). Pressure Plate Studies to Determine How Moisture Affects Access of Bacterial-feeding Nematodes to Food in Soil. Eur. J. Soil Sci..

[83] Atlas of Living Australia *Alyogyne huegelii*: Lilac Hibiscus. https://bie.ala.org.au/species/https://id.biodiversity.org.au/node/apni/2908199.

[84] Atlas of Living Australia *Goodenia ovata*: Hop Goodenia. https://bie.ala.org.au/species/https://id.biodiversity.org.au/node/apni/2898281.

[85] R Core Team (2021). R, Version 4.1.2; A Language and Environment for Statistical Computing.

[86] Fox J., Weisberg S. (2019). An R Companion to Applied Regression.

[87] Lenth R.V. (2023). Emmeans: Estimated Marginal Means, Aka Least-Squares Means, Version 1.8.5.

[88] Cribari-Neto F., Zeileis A. (2010). Beta Regression in R. J. Stat. Softw..

[89] Revelle W. (2024). psych: Procedures for Psychological, Psychometric, and Personality Research, Version 2.4.3.

[90] Hothorn T., Bretz F., Westfall P. (2008). Simultaneous Inference in General Parametric Models. Biom. J..

[91] Wickham H. (2016). ggplot2: Elegant Graphics for Data Analysis.

