# Supplementary material for: Using Recycled Construction Waste Amended with Pine Bark as a Substrate for Urban Plantings
Source: Plants (Basel). 2026 Jan 28;15(3):403. doi: 10.3390/plants15030403 (PMC12899144; doi:10.3390/plants15030403)
Supplement: Supplementary file 1 [file plants-15-00403-s001.zip › plants-4074910-supplementary.pdf]

Supplementary Material

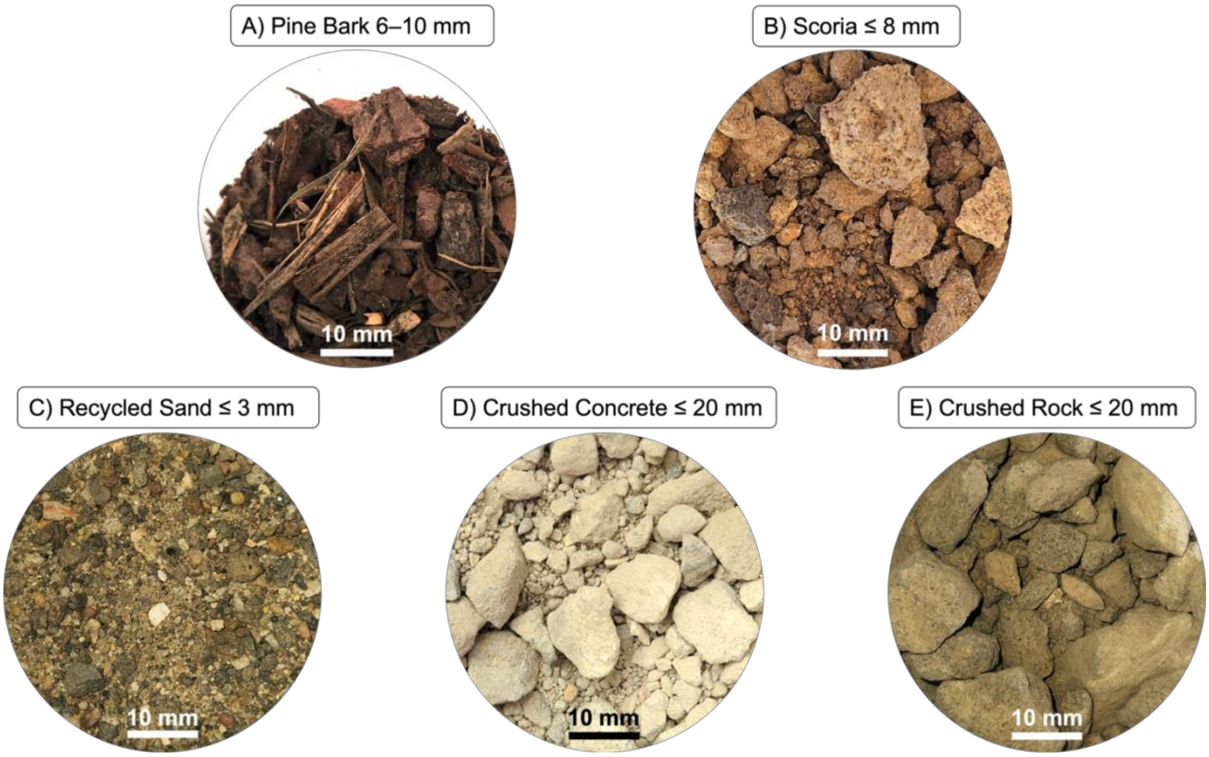

Figure S1: Photographs illustrating the shape and size of particles in the five substrate components. (A) pine bark mulch particles ranged from 6–10mm, were oblong and fibrous, and chocolatey-brown in colour. (B) scoria particles ranged from 8 mm to <0.002 mm, were sub-angular to angular with many vesicles/pores, and reddish-brown in colour. C) Recycled sand particles ranged from 3 mm to <0.002 mm, were a mix of rounded, sub-rounded, and sub-angular, and were brown, grey or black in colour. D) Crushed concrete particles ranged from 20 mm to <0.002 mm, were sub-rounded to sub-angular and light-grey in colour. E) Crushed rock particles ranged from 20 mm to <0.002 mm, were sub-angular to angular dense basalt fragments, grey-brown to black in colour.

Table S1: Two-way ANOVA (type III) for main effects of pine bark addition rate and mineral component and their interaction effect on normally distributed plant response variables (Shoot, Root & Total biomass, RMF, LMF).

Response	Effect	χ^2	D.F.	p-value	Significance
Shoot biomass (g)	Pine bark	30.4	1	< 0.001	***
	Mineral component	26.5	3	< 0.001	***
	Species	264.3	1	< 0.001	***
	Interaction pb:min	13.2	3	0.004	**
Root biomass (g)	Pine bark	6.8	1	0.009	**
	Mineral component	17.6	3	< 0.001	***
	Species	98.8	1	< 0.001	***
	Interaction pb:min	10.3	3	0.017	*
Total biomass (g)	Pine bark	24.6	1	< 0.001	***
	Mineral component	27.5	3	< 0.001	***
	Species	252.5	1	< 0.001	***
	Interaction pb:min	15.1	3	0.002	**
RMF	Pine bark	1.3	1	0.3	
	Mineral component	6.9	3	0.07	
	Species	8.4	1	0.004	**
	Interaction pb:min	3.2	3	0.3	
LMF	Pine bark	1.1	1	0.3	
	Mineral component	0.9	3	0.8	
	Species	372.2	1	< 0.001	***
	Interaction pb:min	0.7	3	0.8	

Significance key: * p < 0.05; ** p < 0.01; *** p < 0.001

Table S2: Pearson correlation of substrate physical, hydrological, and chemical properties

	CI		AFP	PAW	BD	WHC	pH
AFP	0.81	*					
PAW	-0.86	**	-0.62				
BD	-0.36		-0.49	-0.09			
WHC	-0.33		-0.52	0.59	-0.47		
pH	0.23		-0.14	-0.24	0.41	-0.11	
EC	0.20		-0.27	-0.24	0.27	0.19	0.70

Significance key: * p < 0.05; ** p < 0.01; *** p < 0.001

Table S3: Linear regression model output of substrate properties, pine bark addition rate and their interaction effect on total plant biomass separately for *Goodenia ovata and Alyogyne huegelii*. Significant relationships denoted by bold p-values.

Species	Substrate Property	Model Term	Mean Estimate	Std. Error	t value	p-value	R^2	Adjusted R^2	F Statistic	DF1	DF2
Goodenia ovata	Coarseness Index (% w/w)	(Intercept)	201.76	15.13	13.33		0.91	0.85	13.95	3	4
		Pine bark 50%	-95.41	24.54	-3.89	0.02					
		CI	-0.88	0.23	-3.86	0.02					
		CI:Pine Bark 50%	1.00	0.35	2.89	0.04					
	Air Filled Porosity (% v/v)	(Intercept)	158.31	15.39	10.29		0.67	0.41	2.64	3	4
		Pine bark 50%	-47.44	33.35	-1.42	0.23					
		AFP	-1.14	1.17	-0.98	0.38					
		AFP:Pine Bark 50%	1.34	1.82	0.73	0.50					
	Dry Bulk Density (g cm^{-3})	(Intercept)	125.61	49.50	2.54		0.61	0.32	2.09	3	4
		Pine bark 50%	-28.05	71.41	-0.39	0.71					
		BD	15.32	37.29	0.41	0.70					
		BD:Pine Bark 50%	4.44	69.01	0.06	0.95					
	Water Holding Capacity (% v/v)	(Intercept)	142.22	38.95	3.65		0.59	0.29	1.93	3	4
		Pine bark 50%	-8.88	77.77	-0.11	0.91					
		WHC	0.10	1.16	0.09	0.93					
		WHC:Pine Bark 50%	-0.66	2.31	-0.29	0.79					
	Plant Available Water (% w/w)	(Intercept)	119.96	8.37	14.34		0.90	0.83	12.43	3	4
		Pine bark 50%	-2.47	12.02	-0.21	0.85					
		PAW	2.29	0.63	3.62	0.02					
		PAW:Pine Bark 50%	-2.51	0.88	-2.87	0.05					
	pH	(Intercept)	402.61	66.72	6.03		0.92	0.86	14.95	3	4
		Pine bark 50%	-323.33	72.94	-4.43	0.01					
		pH	-27.00	7.00	-3.86	0.02					
		pH:Pine Bark 50%	31.06	7.75	4.01	0.02					
	Electrical Conductivity (dS m^{-1})	(Intercept)	159.40	11.00	14.49		0.77	0.59	4.38	3	4
		Pine bark 50%	-56.54	20.50	-2.76	0.05					
		EC	-43.63	27.14	-1.61	0.18					
		EC:Pine Bark 50%	90.82	68.29	1.33	0.25					

Table S3 (Continued): Linear regression model output of substrate properties, pine bark addition rate and their interaction effect on total plant biomass separately for *Goodenia ovata and Alyogyne huegelii*. Significant relationships denoted by bold p-values.

Species	Substrate Property	Model Term	Mean Estimate	Std. Error	t value	p-value	R^2	Adj. R^2	F Statistic	DF1	DF2
Alyogyne huegelii	Coarseness Index (% w/w)	(Intercept)	119.88	9.00	13.32		0.96	0.92	28.35	3	4
		CI	-0.49	0.14	-3.62	0.02					
		Pine bark 50%	-46.94	14.59	-3.22	0.03					
		CI:Pine Bark 50%	0.30	0.21	1.45	0.2					
	Air Filled Porosity (% v/v)	(Intercept)	102.98	4.37	23.58		0.96	0.93	32.56	3	4
		AFP	-1.30	0.33	-3.91	0.02					
		Pine bark 50%	-32.56	9.47	-3.44	0.03					
		AFP:Pine Bark 50%	0.73	0.52	1.41	0.2					
	Dry Bulk Density (g cm^{-3})	(Intercept)	90.15	30.07	3.00		0.79	0.63	5.04	3	4
		BD	-1.21	22.66	-0.05	0.9					
		Pine bark 50%	-28.35	43.39	-0.65	0.6					
		BD:Pine Bark 50%	-2.17	41.93	-0.05	0.9					
	Water Holding Capacity (% v/v)	(Intercept)	62.15	17.28	3.60		0.88	0.80	10.05	3	4
		WHC	0.81	0.52	1.57	0.2					
		Pine bark 50%	-28.04	34.51	-0.81	0.5					
		WHC:Pine Bark 50%	-0.07	1.02	-0.07	0.9					
	Plant Available Water (% w/w)	(Intercept)	73.04	2.72	26.83		0.99	0.97	87.96	3	4
		PAW	1.39	0.21	6.73	0.003					
		Pine bark 50%	-20.50	3.91	-5.24	0.006					
		PAW:Pine Bark 50%	-0.87	0.29	-3.06	0.04					
	pH	(Intercept)	183.90	74.38	2.47		0.85	0.74	7.66	3	4
		pH	-10.02	7.80	-1.28	0.3					
		Pine bark 50%	-129.55	81.31	-1.59	0.2					
		pH:Pine Bark 50%	10.53	8.64	1.22	0.3					
	Electrical Conductivity (dS m^{-1})	(Intercept)	89.70	8.62	10.41		0.79	0.64	5.07	3	4
		EC	-3.55	21.25	-0.17	0.9					
		Pine bark 50%	-31.66	16.05	-1.97	0.1					
		EC:Pine Bark 50%	6.74	53.47	0.13	0.9					
